# ﻿Three new species of *Camporesiomyces* (Tubeufiaceae, Tubeufiales, Dothideomycetes) associated with coffee in Yunnan, China

**DOI:** 10.3897/mycokeys.117.154573

**Published:** 2025-05-14

**Authors:** Mei-Yan Han, Samantha C. Karunarathna, Li Lu, De-Ge Zheng, Nakarin Suwannarach, Abdallah M. Elgorban, Dong-Qin Dai, Li-Juan Zhang, Wan-Tong Zhao, Ekachai Chukeatirote, Saowaluck Tibpromma

**Affiliations:** 1 Center for Yunnan Plateau Biological Resources Protection and Utilization, College of Biology and Food Engineering, Qujing Normal University, Qujing, Yunnan 655011, China College of Biology and Food Engineering, Qujing Normal University Qujing China; 2 School of Science, Mae Fah Luang University, Chiang Rai, 57100, Thailand School of Science, Mae Fah Luang University Chiang Rai Thailand; 3 Center of Excellence in Fungal Research, Mae Fah Luang University, Chiang Rai 57100, Thailand Center of Excellence in Fungal Research, Mae Fah Luang University Chiang Rai Thailand; 4 Center of Excellence in Microbial Diversity and Sustainable Utilization, Chiang Mai University, Chiang Mai 50200, Thailand Center of Excellence in Microbial Diversity and Sustainable Utilization, Chiang Mai University Chiang Mai Thailand; 5 Office of Research Administration, Chiang Mai University, Chiang Mai 50200, Thailand Office of Research Administration, Chiang Mai University Chiang Mai Thailand; 6 Department of Botany and Microbiology, College of Science, King Saud University, Riyadh, Saudi Arabia King Saud University Riyadh Saudi Arabia

**Keywords:** Coffee-associated microfungi, morphology, multigene phylogeny, taxonomy, three novel species

## Abstract

During our surveys of microfungi associated with coffee plants in Yunnan Province, China, three saprobic fungi were isolated from dead coffee branches. Multigene phylogenetic analyses (ITS, LSU, *tef*1-α, and *rpb*2) and morphological characteristics resulted in the identification of three novel species in *Camporesiomyces*, namely *C.bhatii*, *C.coffeae*, and *C.puerensis*. Detailed morphological descriptions, illustrations, and phylogenetic analyses of these three new species are provided, along with morphological comparisons to closely related taxa. These findings have global implications for understanding the diversity of microfungi associated with coffee trees.

## ﻿Introduction

Coffee (roasted beans of *Coffea*) is one of the most widely consumed beverages globally and ranks as the second most valuable traded commodity after crude oil (Ochoa-Henriquez et al. 2024). Originally from the tropical forests of Ethiopia, coffee is now cultivated globally across equatorial regions, particularly in frost-free areas with adequate rainfall and well-drained soils (Davis et al. 2006). Coffee-producing countries worldwide have their own unique cultural heritage and production methods (Buzzanell 1979). To date, approximately 177 varieties of coffee are available worldwide [Global Biodiversity Information Facility database (GBIF), available at: https://www.gbif.org/species/2895315 (accessed March 4, 2025)]. However, only a few have commercial value due to their moderate caffeine content and distinctive flavor (Wintgens, 2012). *Coffeaarabica* and *Co.canephora* are the most widely cultivated coffee species, with *Co.arabica* accounting for approximately 60–70% and *Co.canephora* around 30–40% of global coffee production. In comparison, *Co.liberica* is grown in a few countries and contributes less than 1% (Bilen et al. 2022). *Coffeaarabica* is prized for its mild flavor and aromatic complexity, while *Co.eugenioides*, a progenitor of *Co.arabica*, contributes genetic diversity critical for breeding programs (Scalabrin et al. 2020). *Coffeacanephora* is valued for its higher caffeine content and its resilience to pests and climatic fluctuations (Davis et al. 2006; Avelino et al. 2015). *Coffealiberica*, is a plant of the humid, lowland tropics (Soerianegara and Lemmens 1993), characterized by its large beans and resistance to certain diseases (Melia et al. 2025).

The history of coffee cultivation in China can be traced back to 1884, when British merchants first introduced coffee to Taiwan Province of China. The earliest coffee cultivation on mainland China began in the early 20^th^ century when French missionaries brought coffee seedlings to Binchuan County, Yunnan Province, for cultivation (Zhang et al. 2014). Coffee production in Yunnan Province accounts for approximately 90% of China’s total coffee production (Neilson and Wang 2019). Pu’er City is one of the largest coffee production areas in China, with the largest planting area and output. It primarily grows Arabica coffee, which enjoys a high reputation in the international market for its unique flavor and high quality (Li 2014). Xishuangbanna Autonomous Prefecture is also one of China’s important coffee-producing areas. Coffee planting there is mainly concentrated in Jinghong City, Menghai County, and Mengla County (Rigal et al. 2018).

Microfungi play a crucial role in agricultural ecosystems and are key nutrient regulating organisms in coffee ecosystems. They are globally distributed and exhibit high diversity (Bahram & Netherway, 2022). In recent years, scientists have conducted in-depth research on coffee-related fungi, identifying approximately 600 species (Lu et al. 2022b; Poma-Angamarca et al. 2024). However, relatively less attention has been paid to saprophytic fungi associated with coffee, with only more than 60 species reported globally to date (Han et al. 2024; Lu et al. 2025). Coffee-associated saprophytic fungi play a crucial ecological role in ecosystems and have also attracted researchers’ interest in recent years due to their potential for biocontrol (Lu et al. 2022a). For example, Rodríguez et al. (2016) demonstrated that coffee saprobes, as decomposers, significantly reduce anthracnose incidence in *Coffeaarabica* seedlings through pathogen suppression, indicating their potential as biocontrol agents. In addition, Rodríguez et al. (2024) showed that certain yeast strains isolated from Robusta coffee significantly inhibit the growth of *Aspergilluscarbonarius* and the production of ochratoxin A (OTA). These strains demonstrated an 85% inhibition rate of pathogen growth and a 90% toxin degradation efficiency *in vitro*. Thus, investigating the diversity of coffee saprophytic fungi is crucial, as it lays the groundwork for future studies exploring their potential applications in sustainable coffee farming.

Tubeufiales was established by Boonmee et al. (2014) based on phylogenetic analyses and morphological characteristics to accommodate the monophyletic family Tubeufiaceae, which has previously been classified in Pleosporales. Later, two additional families, Bezerromycetaceae and Wiesneriomycetaceae were incorporated into Tubeufiales by Liu et al. (2017) based on divergence time estimates. The latest comprehensive revision of Tubeufiales was conducted by Lu et al. (2018b), who expanded the circumscription of the type family Tubeufiaceae and revised the taxonomy of tubeufiaceous species. This revision notably helped highlight cases of misidentified fungi and determine which morphological characters are crucial for classifying species accurately. Members of Tubeufiales (Bezerromycetaceae, Tubeufiaceae, and Wiesneriomycetaceae) exhibit a widespread distribution, ranging from temperate to tropical regions (Boonmee et al. 2014; Liu et al. 2017; Xu et al. 2022; Hyde et al. 2024), and are commonly found on decorticated or decaying woody and herbaceous substrates as saprobic fungi. They often coexist with other decaying fungi (Lu et al. 2018b).

Tubeufiaceae was established by Barr (1979) based on the type genus *Tubeufia* to accommodate some bitunicate ascomycetes that grow on decaying wood. Members of Tubeufiaceae are widely distributed in tropical, subtropical, and temperate regions, and are often found on woody substrates in both terrestrial and freshwater habitats (Boonmee et al. 2011, 2014; Luo et al. 2017; Lu et al. 2018b, Tibpromma et al. 2018; Ma et al. 2024). A total of 47 genera are listed in Tubeufiaceae (Hyde et al. 2024), with *Tubeufia* being the largest genus. In addition, some studies have demonstrated that members of the Tubeufiaceae can produce biologically active secondary metabolites with antifungal, antibacterial, antidiabetic, and anticancer properties (Itazaki et al. 1990; Hanada et al. 1996; Hu et al. 2006; Lu et al. 2018; Zhang et al. 2023; Zhang et al. 2024).

Based on integrated morphological and phylogenetic analyses of combined ITS, LSU, *tef*1-α, and *rpb*2 sequences, *Camporesiomyces* was proposed by Hyde et al. (2020) to accommodate a new species, *C.mali* (type species), and two new combinations, *C.patagonicus* and *C.vaccinii* (formerly belonging to the genera *Acanthostigma* and *Helicoma*, respectively) (Carris 1989; Sanchez et al. 2012). Morphologically, the type species, *C.mali*, is distinguished from other members of Tubeufiaceae by its unique multi-loculate ascomata and narrowly fusiform, hyaline, multi-septate ascospores (Hyde et al. 2020). The asexual morph of *Camporesiomyces* is characterized by monoblastic or polyblastic conidiogenous cells, which are denticulate, with helical and hyaline conidia that are smooth-walled (Carris 1989). *Camporesiomycespatagonicus* and *C.vaccinii* were previously classified as *Acanthostigmapatagonicum* and *Helicomavaccinii*, respectively (Carris 1989; Sanchez et al. 2012). However, the natural classification of these two taxa has been uncertain (Boonmee et al. 2014; Lu et al. 2018). Although the holotypes of *A.patagonicum* and *H.vaccinii* have not been rechecked, the DNA data derived from the ex-type strains show they belong to *Camporesiomyces* (Hyde et al. 2020). Currently, the genus comprises only three species (*C.mali*, *C.patagonicus*, and *C.vaccinii*), all of which exhibit consistent morphological autapomorphies and molecular divergence that warrant their segregation from allied genera (Hyde et al. 2020).

In this study, we aim to introduce three new species of *Camporesiomyces* found on dead coffee branches collected from Pu’er and Xishuangbanna, Yunnan Province, China. Morphological characteristics and multi-locus phylogeny analyses, based on combined ITS, LSU, *tef*1-*α*, and *rpb*2 sequence data, were used to confirm the taxonomic placement of the new species.

## ﻿Materials and methods

### ﻿Specimen collection, examination, and isolation

Dead branches of Arabica Coffee and Liberica Coffee with fungal fruiting bodies were collected from Pu’er City and Xishuangbanna Autonomous Prefecture, Yunnan Province, China. The specimens were placed in self-sealing bags, with sampling information recorded on the cover (Rathnayaka et al. 2025) and transported to the mycology laboratory at Qujing Normal University. The fruiting body structures were observed using a LEICA S8 APO optical microscope (Olympus, Tokyo, Japan). Micro-morphological characteristics were observed with a compound microscope (OLYMPUS BX53, Olympus, Tokyo, Japan), and photographed with an OLYMPUS DP74-CU camera fitted onto the microscope. Measurement was made using the Tarosoft (R) Image Framework v. 0.9.7 program. The photo plate was created in Adobe Photoshop CS3 Extended version 10.0 software (Adobe Systems, USA). Single spore isolation was performed following the method outlined by Senanayake et al. (2020) to obtain pure cultures on potato dextrose agar (PDA). Dried herbarium samples were preserved at the Herbarium of Guizhou Medical University, Guiyang, China (GMB-W). Living cultures were deposited in the Guizhou Medical University Culture Collection (GMBCC), Guiyang. The Facesoffungi (FoF) and Index Fungorum (IF) numbers were obtained as explained in Jayasiri et al. (2015) and Index Fungorum (2025), respectively.

### ﻿DNA extraction, PCR amplification, and sequencing

Fresh mycelia grown on PDA (15–30 days) were scraped and transferred to 1.5 ml microcentrifuge tubes for DNA extraction. The Biospin Fungus Genomic DNA Extraction Kit BSC14S1 (BioFlux, China) was used to obtain DNA products from the above mycelia, according to the manufacturer’s protocol. The extracted DNA was then preserved at -20 °C for future use. Polymerase chain reaction (PCR) was performed for four loci; the details of the different loci, primers, and PCR thermal cycle programs used for amplification are presented in Table 1. PCR amplification was performed in a reaction volume of 25 µL, consisting of 12.5 µL of 2× Bench Top™ Taq Master Mix, 8.5 µL of ddH_2_O, 2 µL of each forward and reverse primer, and 2 µL of the DNA template. The PCR products were purified and sequenced by Sangon Biotechnology Co., Ltd. (Shanghai, China). All newly generated sequences in this study were deposited in GenBank (http://www.ncbi.nlm.nih.gov/genbank/) and are listed in Table 2.

**Table 1.** Loci, primers, and amplification procedure used in this study.

Loci	Primers	PCR conditions	References
ITS	ITS5/ITS4	94 °C: 3 mins, (94 °C: 45 s, 55 °C: 50 s, 72 °C: 1 min) × 35 cycles, 72 °C: 10 mins	White et al. (1990)
LSU	LR5/LR0R		Vilgalys and Hester (1990)
*tef*1-α	983F/2218R		Rehner and Samuels (1994)
*rpb*2	5F/7cR	95 °C: 5 mins, (95 °C: 1 min, 55 °C: 2 min, 72 °C: 90 s) × 40 cycles, 72 °C: 10 mins	Liu et al. (1999)

**Table 2.** Names, strain numbers, and corresponding GenBank accession numbers of the taxa used in the phylogenetic analyses. The newly generated sequences are in bold black. After the strain number, “T” indicates the type strains. “NA” indicates sequence unavailability.

Fungal species	Strain numbers	ITS	LSU	*tef*1-α	*rpb*2
*Acanthohelicosporaaurea*	GZCC 16-0059	KY321322	KY321325	KY792599	MF589910
*Acanthohelicosporaaurea*	GZCC 16-0060	KY321323	KY321326	KY792600	MF589911
*Acanthostigmachiangmaiense*	MFLUCC 10-0125^T^	JN865209	JN865197	KF301560	NA
*Acanthostigmamultiseptatum*	ANM 475^T^	GQ856145	GQ850492	NA	NA
*Acanthostigmamultiseptatum*	ANM 665	GQ856144	GQ850493	NA	NA
*Acanthostigmaperpusillum*	UAMH 7237^T^	AY916492	AY856892	NA	NA
*Acanthostigmascopulum*	ANM 95	GQ856142	GQ850490	NA	NA
*Acanthostigmascopulum*	ANM 386	GQ856141	GQ850489	NA	NA
*Berkleasmiumfusiforme*	MFLUCC 17-1978^T^	MH558693	MH558820	MH550884	MH551007
*Berkleasmiumfusiforme*	MFLUCC 17-1979	MH558694	MH558821	MH550885	MH551008
*Bezerromycespernambucoensis*	URM7414	KX470393	KX518626	KX518634	NA
*Bezerromycespernambucoensis*	URM7412	KX470391	KX518624	KX518632	NA
***Camporesiomycesbhatii***	**GMBCC 1120^T^**	**PQ763360**	**PQ842543**	**PV388888**	**PV388894**
***Camporesiomycesbhatii***	**GMBCC 1125**	**PQ763361**	**PQ842544**	**PV388889**	**PV388895**
***Camporesiomycescoffeae***	**GMBCC 1130^T^**	**PQ763358**	**PQ842545**	**PV388890**	**PV388896**
***Camporesiomycescoffeae***	**GMBCC 1131**	**PQ763359**	**PQ842546**	**PV388891**	**PV388897**
*Camporesiomycesmali*	KUMCC 19-0216^T^	NR_169709	NG_075312	MN794018	NA
*Camporesiomycespatagoniensis*	BBB MVB 573	JN127358	JN127359	NA	NA
***Camporesiomycespuerensis***	**GMBCC 1113^T^**	**PQ763356**	**PQ842541**	**PV388886**	**PV388892**
***Camporesiomycespuerensis***	**GMBCC 1114**	**PQ763357**	**PQ842542**	**PV388887**	**PV388893**
*Camporesiomycesvaccinii*	CBS 216.90	MH862204	MH873889	NA	NA
*Chlamydotubeufiacylindrica*	MFLUCC 16-1130^T^	MH558702	MH558830	MH550893	MH551018
*Chlamydotubeufiahuaikangplaensis*	MFLUCC 16-0023	KY678766	KY678758	KY792597	MF535259
*Dematiohelicosporumguttulatum*	MFLUCC 17-2011^T^	MH558705	MH558833	MH550896	MH551021
*Dictyosporathailandica*	MFLUCC 16-0001^T^	KY873627	KY873622	KY873286	MH551023
*Dictyosporathailandica*	MFLUCC 18-0641	MH558706	MH558834	MH550897	MH551022
*Helicoarctatusaquaticus*	MFLUCC 17-1996^T^	MH558707	MH558835	MH550898	MH551024
*Helicomamuelleri*	CBS 964.69^T^	AY916453	MH871278	NA	NA
*Helicomanematosporum*	MFLUCC 16-0011	MH558722	MH558848	MH550913	MH551039
*Helicomarubriappendiculatum*	MFLUCC 18-0491^T^	MH558723	NG_069593	MH550914	MH551040
*Helicomyceshyalosporus*	GZCC 16 0070	MH558728	MH558854	MH550919	MH551044
*Helicomycestorauatus*	MELUCC16-0217	MH558732	MH558858	MH550923	MH551048
*Helicosporiumnanningense*	GZCC 22-2175^T^	OR066418	OR066425	OR058864	OR058857
*Helicosporiumnanningense*	GZCC 23-0588	OR066419	OR066426	OR058865	OR058858
*Helicosporiumsetiferum*	MFLUCC 17-1994^T^	MH558735	MH558861	MH550926	MH551051
*Helicosporiumsetiferum*	MFLUCC 17-2007	MH558737	MH558863	MH550928	MH551053
*Helicosporiumsexuale*	GZCC 22-2007	OP508731	OP508771	OP698082	OP698071
*Helicosporiumsexuale*	MFLUCC 16-1244	MZ538503	MZ538537	MZ567082	MZ567111
*Helicosporiumvegetum*	BCC 8125	AY916491	MH871277	NA	NA
*Helicosporiumvegetum*	CBS 254.75	NA	DQ470982	DQ471105	NA
*Helicosporiumvegetum*	CBS 941.72^T^	AY916488	AY856883	NA	NA
*Helicosporiumvesicarium*	MFLUCC 17-1795^T^	MH558739	MH558864	MH550930	MH551055
*Helicosporiumviridiflavum*	MFLUCC 17-2336^T^	MH558738	NA	MH550929	MH551054
*Helicotubeufiaguangxiensis*	MFLUCC 17-0040^T^	MH290018	MH290024	MH290029	MH290034
*Helicotubeufiahydei*	MFLUCC 17-1980^T^	MH290021	MH290026	MH290031	MH290036
*Kamalomycesthailandicus*	MFLUCC 13-0233^T^	MF506884	MF506882	MF506886	NA
*Kamalomycesthailandicus*	MFLUCC 11-0158	MF506883	MF506881	MF506885	MF506887
*Neoacanthostigmafusiforme*	MFLUCC 11-0510^T^	KF301529	KF301537	NA	NA
*Neohelicomycesaquaticus*	KUMCC 15-0463	KY320529	KY320546	KY320562	MH551065
*Neotubeufiakrabiensis*	MFUCC 16-1125^T^	MG012031	MG012024	MG012010	MG012017
*Thaxteriellainthanonensis*	MFLUCC 11-0003^T^	JN865211	JN865199	NA	NA
*Thaxteriellopsislignicola*	MFLUCC 16-0026	MH558768	MH558893	MH550960	MH551094
*Thaxteriellopsislignicola*	MELUCC 16-0024	MH558767	MH558892	MH550959	MH551093
*Tubeufiabambusicola*	MFLUCC 17-1803^T^	MH558771	MH558896	MH550963	MH551097
*Tubeufiajavanica*	MFLUCC 12-0545^T^	KJ880034	KJ880036	KJ880037	NA

### ﻿Phylogenetic analyses

The assembly of the forward and reverse primers for the recently obtained sequence was accomplished using BioEdit version 7.0.5.3 and SeqMan version 7.0.0 software packages (DNASTAR, Madison, WI) (Plasterer 1997; Hall 1999). For phylogenetic analysis, relevant sequences were sourced from GenBank based on BLASTn search results and in accordance with the most current literature (Tanaka et al. 2017; Hyde et al. 2018). The sequence data were aligned using the online multiple alignment program MAFFT version 7 (https://mafft.cbrc.jp/alignment/server/) (Katoh and Standley 2013) to ensure that all sequences were in the correct orientation. The alignments were automatically adjusted using trimAL v1.2 (Capella-Gutiérrez et al. 2009) and manually optimized with BioEdit v7.0.5.3 (Hall 1999). The multiple gene sequences were combined using the SequenceMatrix-Windows-1.7.8 software. The aligned multigene sequences were transformed into the PHYLIP format for Maximum Likelihood (ML) analysis and the NEXUS format for Bayesian Inference (BI) using the online utility ALTER (ALignment Transformation EnviRonment) (Glez-Peña et al. 2010). Maximum likelihood (ML) and Bayesian inference (BI) analyses were performed using the online CIPRES Science Gateway platform (https://www.phylo.org/portal2/home.action) (Dissanayake et al. 2020; Ma et al. 2023). The ML tree was constructed using the RAxML-HPC v.8 tool on XSEDE (version 8.2.12) (Stamatakis 2006) with the GTRGAMMA model and 1,000 bootstrap pseudoreplicates. The BI was carried out using the tool MrBayes on XSEDE (3.2.7a) (Huelsenbeck and Ronquist 2001; Stamatakis et al. 2008; Ronquist et al. 2012), using the Markov Chain Monte Carlo (MCMC) method, six simultaneous Markov chains were run for 2,000,000 generations, and trees were sampled at every 100^th^ generation. Prior to conducting BI, the model of evolution for each gene region was estimated using MrModelTest version 2 (Ma 2016), The phylogenetic trees were edited by FigTree v. 1.4.0 (http://tree.bio.ed.ac.uk/software/figtree/).

## ﻿Results

### ﻿Phylogenetic analyses

**Figure 1.**
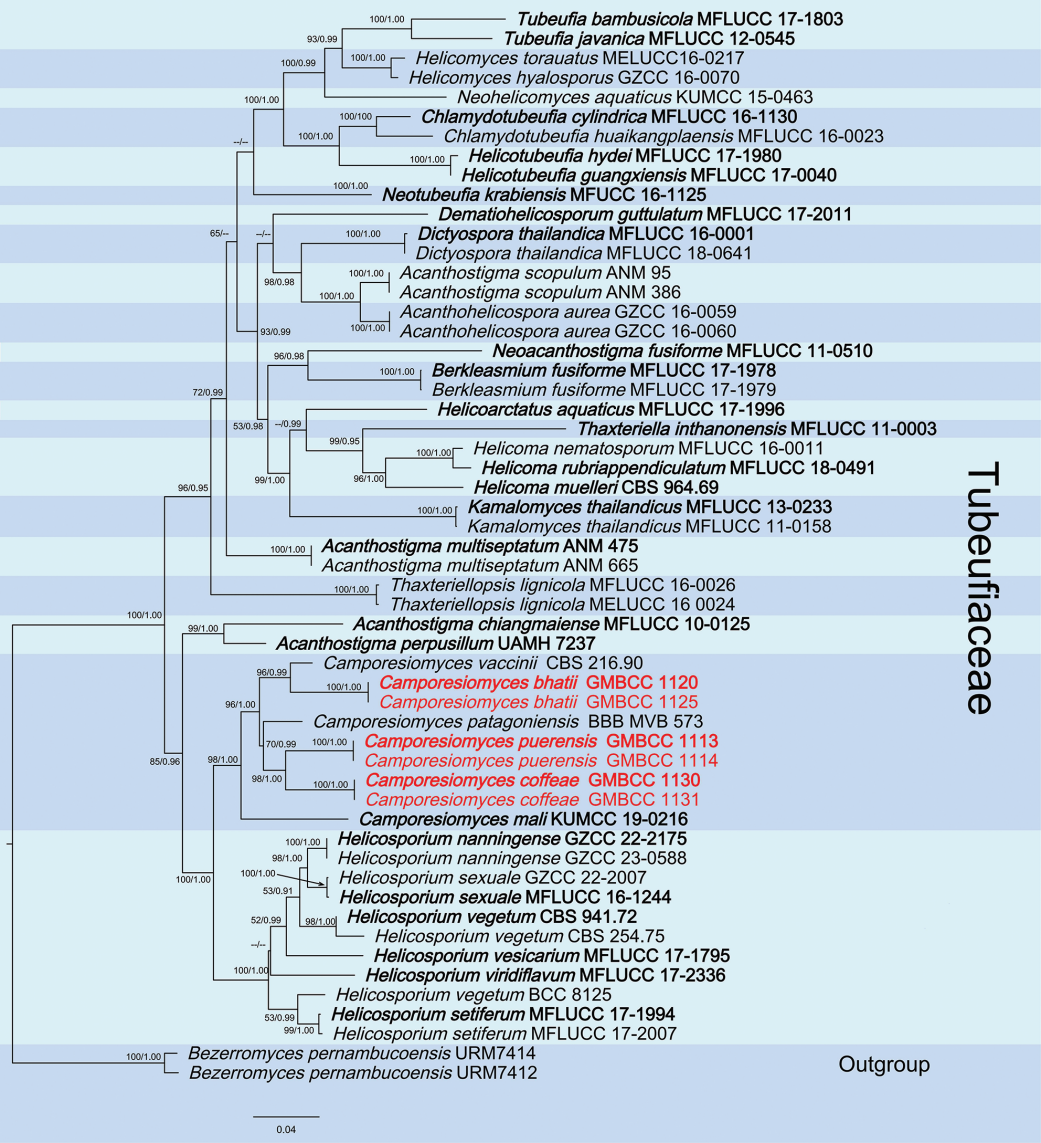
The ML analysis and Bayesian inference (BI) analyses yielded nearly identical tree topologies, with bootstrap support values for ML equal to or greater than 50%, and BI analysis values equal to or greater than 0.90 PP are provided at each node. Newly generated sequences are in red, while the ex-type strains are in bold.

Three new species (*Camporesiomycesbhatii*, *C.coffeae*, and *C.puerensis*) formed a distinct clade within *Camporesiomyces*, with strong statistical support (ML = 100%, PP = 1.00). *Camporesiomyces* clade was sister to *Helicosporium* within the family Tubeufiaceae, and all genera in Tubeufiaceae are distinctly separated (Fig. 1). The RAxML tree was based on a combined dataset of ITS, LSU, *tef*1-α, and *rpb*2 gene sequence data, which comprised 3,255 characters (ITS:1– 437, LSU: 438–1257, *rpb2*: 1258–2357, *tef1-α*: 2358–3255), including gaps. *Bezerromycespernambucoensis* (URM7414) and *B.pernambucoensis* (URM7412) were used as outgroup taxa. The topology of the phylogenetic tree constructed using the maximum likelihood (ML) method exhibited a high degree of similarity to that generated by Bayesian inference (BI). Consequently, the ML tree was selected to illustrate the evolutionary history of *Camporesiomyces*.

The best-scoring RAxML tree, with a final ML optimization likelihood value of -26167.982465 is presented. The matrix contained 1,266 distinct alignment patterns, with 21.89% of the characters being undetermined or gaps. Estimated base frequencies were as follows: A = 0.243800, C = 0.255403, G = 0.265360, T = 0.235437; substitution rates: AC = 0.936178, AG = 5.168669, AT = 2.168333, CG = 0.811291, CT = 10.165688, GT = 1.000000; gamma distribution shape parameter *α* = 0.816594. The phylogenetic tree resulting from RAxML analysis is shown in Fig. 1.

According to the phylogenetic tree, our three new species (*Camporesiomycesbhatii*, *C.coffeae*, and *C.puerensis*) stand out as distinct entities within the genus *Camporesiomyces*. Among these, *C.bhatii* (GMBCC 1120 (ex-type) and GMBCC 1125) formed a sister lineage to *C.vaccinii* (CBS 216.90) with 96% ML/0.99 PP support. The other two new species, *C.puerensis* (GMBCC 1113 (ex-type) and GMBCC 1114) and *C.coffeae* (GMBCC 1130 (ex-type) and GMBCC 1131), clustered together but formed well-separated branches (98% ML/1.00 PP support), further emphasizing their distinctiveness. These two new species create a distinct branch that diverges from *C.patagoniensis* (BBB MVB 573) with 70% ML/0.99 PP support.

### ﻿Taxonomy

#### ﻿ Camporesiomyces bhatii

Taxon classificationFungiTubeufialesTubeufiaceae

M.Y. Han & Tibpromma sp. nov.

3AE3B61B-1D1E-5CD5-81E4-F2852E6F0B36

Index Fungorum: IF903588

Facesoffungi Number: FoF17675

Fig. 2

##### Etymology.

The species epithet “*bhatii*” honors Prof. Jayarama Darbhe Bhat for his immense contributions to mycology.

**Figure 2.**
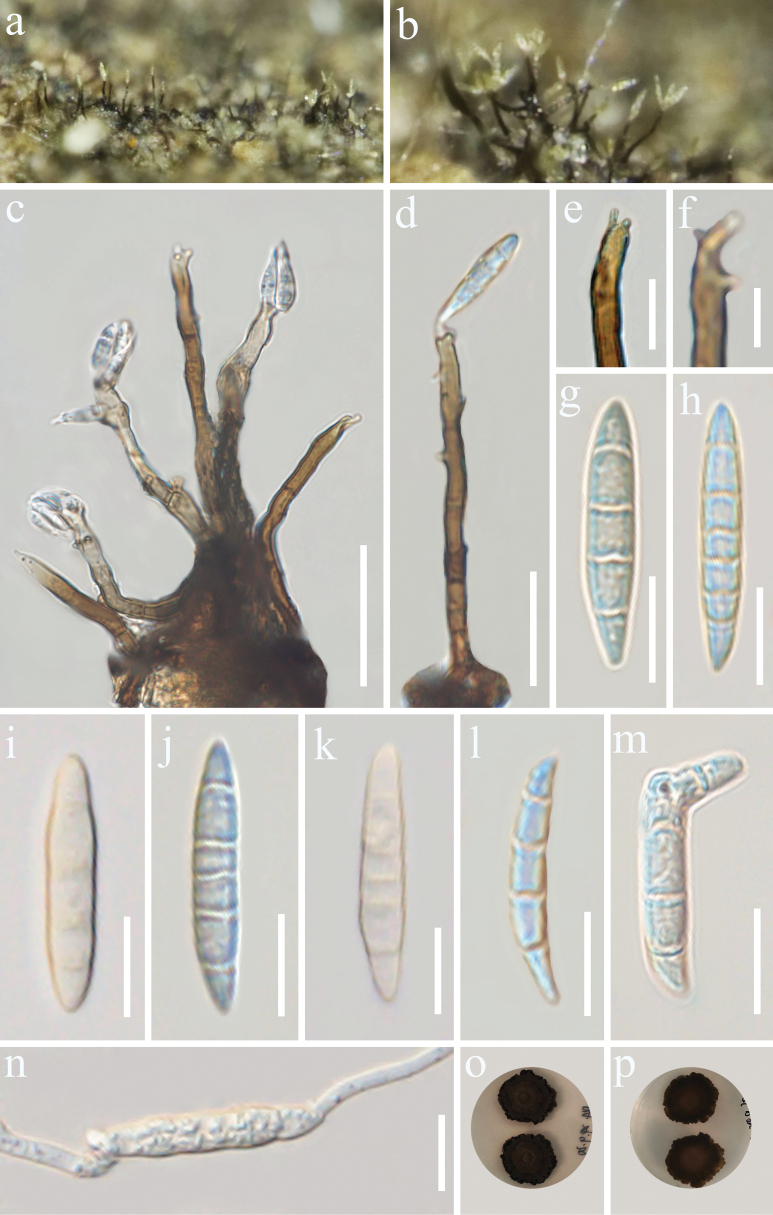
*Camporesiomycesbhatii* (GMB-W1176, holotype) **a, b** colonies on the natural substrate **c, d** conidiophore with conidia and conidiogenous cells **e, f** conidiogenous cells **g–m** conidia **n** a germinated conidium **o, p** Culture on PDA from above and reverse. Scale bars: 50 μm (**c**); 20 μm (**d**); 10 μm (**e**, **g–n**); 5 μm (**f**).

##### Holotype.

GMB-W1176

##### Description.

***Saprobic*** on dead branches of *Coffealiberica*. **Sexual morph**: Undetermined. **Asexual morph**: Hyphomycetous. *Colonies* on natural substrate superficial, solitary or in clusters, hairy, yellow at apex, velvety. Mycelium exposed on the surface of substrate, except on the roots. ***Conidiophores*** 23–87.4 × 2.4–5.7 μm (x̄ = 63 × 3.7 μm, *n* = 30) macronematous, mononematous, either solitary or forming small clusters, smooth or occasionally verruculose, cylindrical, brown, slightly flexuous, simple, unbranched, 2–8-septate, sometimes slightly constricted at the septa. ***Conidiogenous cells*** 8.8–21.7 × 3–4.7 μm (x̄ = 17 × 3.5 μm, *n* = 20), polyblastic, terminal, cylindrical, slightly tapering, conspicuously denticulate on conidial secession, pale brown. ***Conidia*** 16–30 × 3.3–6.3 μm (x̄ = 24 × 6.5 μm, *n* = 30), solitary, acrogenous, cylindrical, obclavate or fusiform, 3–8-septate, subhyaline to pale brown.

##### Culture characteristics.

Conidia germinate on PDA within 24 h at 28 °C, colony on PDA reaching 3 cm diam. after two weeks, circular or irregular, umbonate, with the entire margin dark brown to black. The reverse side displays predominantly black colonies with brown peripheral edges.

##### Material examined.

China • Yunnan Province, Xishuangbanna Autonomous Prefecture (21°41'N, 101°25'E 570 m), on dead branches of *Coffealiberica*, 27 August 2024, M.Y. Han & Tibpromma, (DL4 = GMB-W1176, holotype), ex-type GMBCC 1120, other living culture GMBCC 1125.

##### GenBank number.

GMBCC 1120 = ITS: PQ763360, LSU: PQ842543, *tef*1-α: PV388888, *rpb*2: PV388894 and GMBCC 1125 = ITS: PQ763361, LSU: PQ842544, *tef*1-α: PV388889, *rpb*2: PV388895.

##### Notes.

In the phylogenetic tree, our collection *Camporesiomycesbhatii* [GMBCC 1120 (ex-type) and GMBCC 1125] formed a well-separated lineage, sister to *C.vaccinii* [CBS 216.90, ex-type] with 96% ML/0.99 PP support (Fig. 1). ITS and LSU gene sequences blast results showed that our strain showed 94.14% and 99.23% similarities to *C.vaccinii* (MH862204) and (MH873889), respectively (Carris 1989). In the morphological comparison between *C.bhatii* and *C.vaccinii*, significant differences are observed in both conidial and conidiophore dimensions, as well as conidial shape (Carris 1989). The conidia of *C.bhatii* are notably longer than those of *C.vaccinii* (16–30 × 3.3–6.3 μm) compared to (8.0–13.0 × 2.0–4.0 μm). Conversely, the conidiophores of *C.bhatii* are shorter than those of *C.vaccinii* (23–87.4 × 2.4–5.7 μm) compared to (64–145 × 4.2–5.0 μm) (Carris 1989). Additionally, the conidia of *C.bhatii* exhibit complex shapes, such as obclavate or fusiform, and are subhyaline to pale brown. In contrast, the conidia of *C.vaccinii* are spirally coiled and hyaline or pale brown (Carris 1989). These distinct morphological characteristics provide a clear basis for differentiating the two species. Therefore, based on molecular data and morphological comparison, *C.bhatii* was identified as a new species.

#### ﻿ Camporesiomyces coffeae

Taxon classificationFungiTubeufialesTubeufiaceae

M.Y. Han &Tibpromma sp. nov.

33038255-9CC8-5566-B67C-A3FC2D18153F

Index Fungorum: IF903589

Facesoffungi Number: FoF17676

Fig. 3

##### Etymology.

The species epithet “*coffeae*” refers to the host genus *Coffea*.

**Figure 3.**
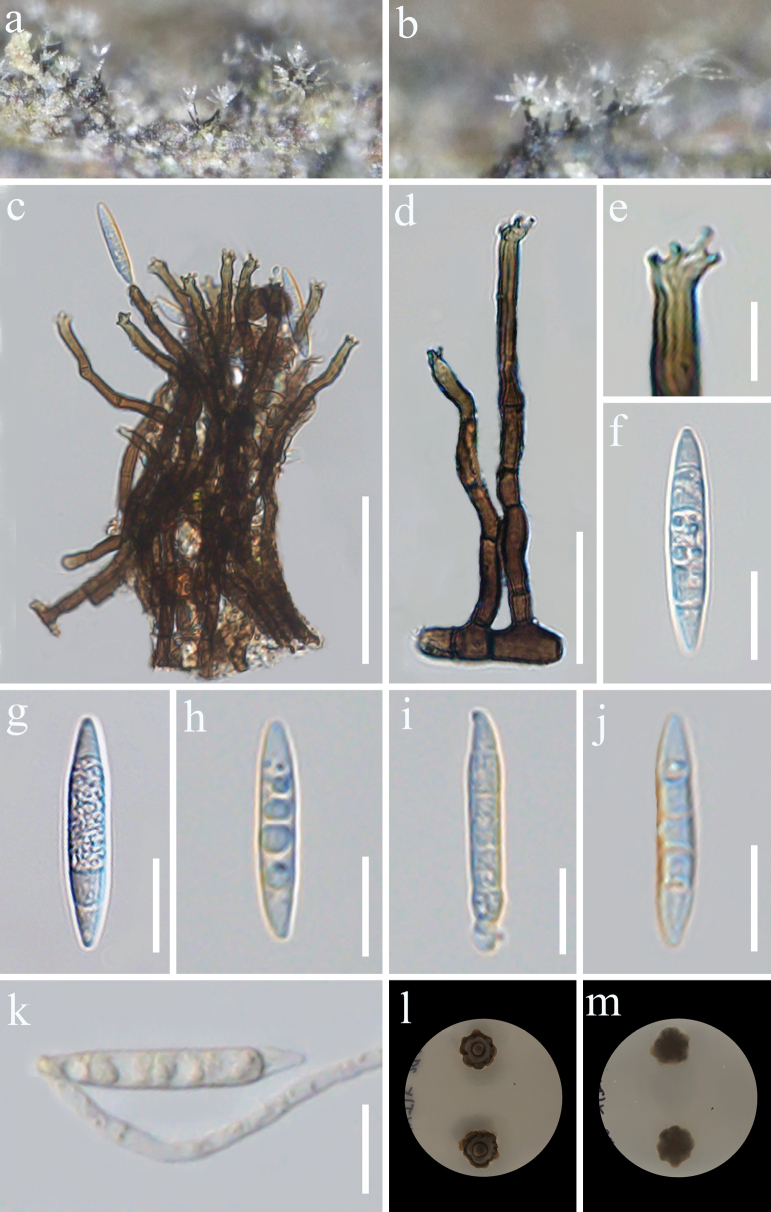
*Camporesiomycescoffeae* (GMB-W1181, holotype) **a, b** colonies on the natural substrate **c, d** conidiophore and conidiogenous cells **e** conidiogenous cells **f–j** conidia **k** a germinated conidium **l, m** culture on PDA from above and reverse. Scale bars: 50 μm (**c**); 20 μm (**d**); 5 μm (**e**); 10 μm (**f**–**k**).

##### Holotype.

GMB-W1181

##### Description.

***Saprobic*** on dead branches of *Coffeaarabica*. **Sexual morph**: Undetermined. **Asexual morph**: Hyphomycetous. *Colonies* on natural substrate superficial, solitary or clusters, hairy, subhyaline at apex, bunch of flowers-like, shiny. Mycelium exposed on surface of substrate, except on the roots. ***Conidiophores*** 43–97 × 2.8–4.5 μm (x̄ = 68 × 3.7 μm, *n* = 30) macronematous, mononematous, either solitary or forming small clusters, smooth or occasionally verruculose, cylindrical, dark brown, slightly flexuous, simple, unbranched, with longitudinal striations in the upper part, 2–9-septate, sometimes slightly constricted at the septa. ***Conidiogenous cells*** 8–21 × 2.3–4.6 μm (x̄ = 15.4 × 3.4 μm, *n* = 30), polyblastic, terminal, cylindrical, with longitudinal striations, with several conspicuous denticles at apex, brown. ***Conidia*** 20–50 × 3.3–6.5 μm (x̄ = 26.7 × 4.5 μm, *n* = 30), solitary, acrogenous, guttules, cylindrical, fusiform, 3–7-septate, subhyaline or hyaline, obtuse or conical at both ends.

##### Culture characteristics.

Conidia germinating on PDA within 24 h at 28 °C, colony on PDA reaching 2 cm diam. after two weeks, circular or irregular, umbonate, with entire margin dark grey to black, irregular, the reverse side displays predominantly black colonies with brown peripheral edges.

##### Material examined.

China • Yunnan Province, Pu’er City, Simao District (22°35'53"N, 100°59'17"E, 1186.4 m), on dead branches of *Coffeaarabica*, 8 August 2024, M.Y. Han & Tibpromma, (YYT15 = GMB-W1181, holotype), ex-type GMBCC 1130; other living culture GMBCC 1131.

##### GenBank number.

GMBCC 1130 = ITS: PQ763358 LSU: PQ842545*tef*1-α: PV388890, *rpb*2: PV388896 and GMBCC 1131 = ITS: PQ763359 LSU: PQ842546*tef*1-α: PV388891, *rpb*2: PV388897.

##### Notes.

**Figure 4.**
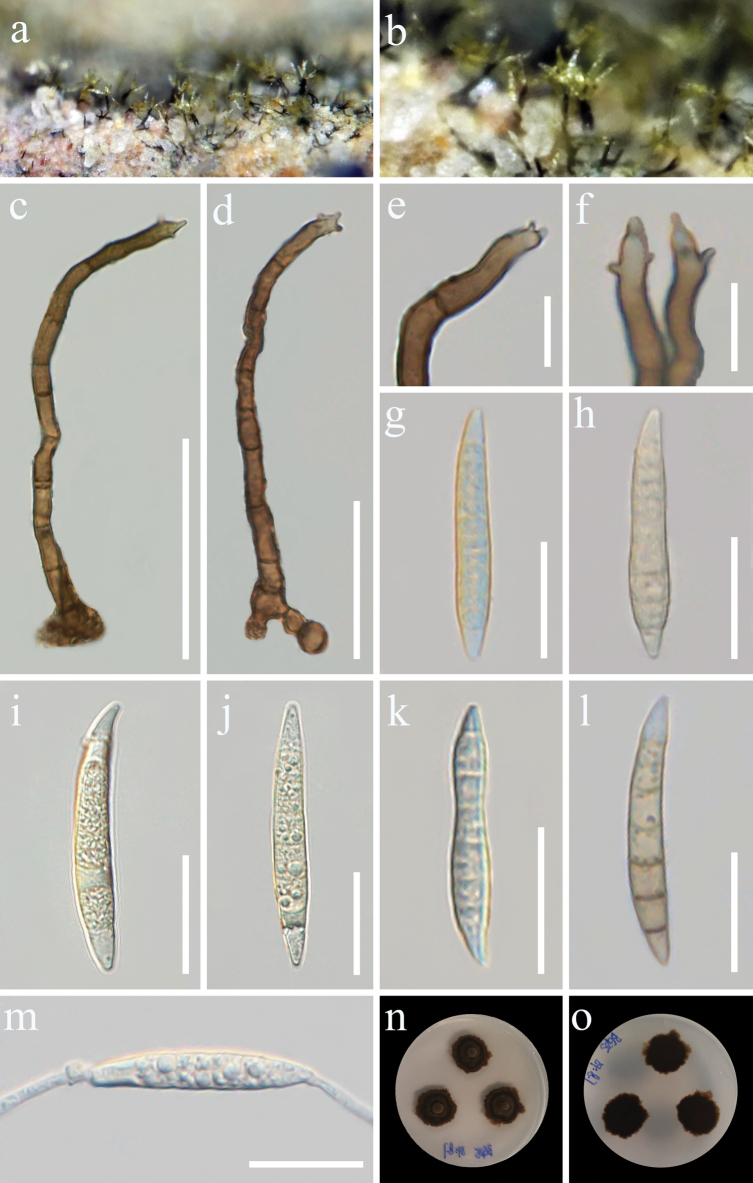
*Camporesiomycespuerensis* (GMB-W1121, holotype) **a, b** colonies on the natural substrate **c, d** conidiophores and conidiogenous cells **e, f** conidiogenous cells **g–l** conidia **m** a germinated conidium **n, o** culture on PDA from above and reverse. Scale bars: 50 μm (**c**); 30 μm (**d**); 10 μm (**e, f**); 20 μm (**g**–**m**).

Based on multi-gene phylogenetic analysis, *Camporesiomycescoffeae* [GMBCC 1130 (ex-type) and GMBCC 1131] is a distinct species, sister to *C.puerensis* [GMBCC 1113 (ex-type) and GMBCC 1114] with 98% ML/1.00 PP support (Fig. 1). Comparative genomic analysis demonstrated that the sequence similarity between *C.coffeae* and *C.puerensis* was 93.00% in the ITS locus, 98.33% in the LSU locus, 93.34% in the *tef*1-α locus, and 89.03% in the *rpb*2 locus (Table. 3). Morphologically, *C.coffeae* can be distinguished from *C.puerensis* by the dimensions and septation of conidiophores and conidia, in that the conidia and conidiophores of *C.coffeae* are smaller than *C.puerensis* (20–50 × 3.3–6.5 μm vs. 21.7–83 × 4–9.4 μm) and (43–97 × 2.8–4.5 μm vs. 52–176.5 × 2.8–5.6 μm), respectively (Figs 3, 4). In addition, the conidia of *C.coffeae* are subhyaline or hyaline, whereas those of *C.puerensis* are hyaline at their ends but yellow in the middle. Moreover, the conidiophores of *C.coffeae* are distinguished by longitudinal striations in their apical sections, a feature that is absent in *C.puerensis*. *Camporesiomycescoffeae* is similar to *C.puerensis* in having polyblastic, terminal, conidiogenous cells, which are brown (Figs 3, 4). Based on the differences between the two species, we describe *C.coffeae* as a new species.

**Table 3.** Nucleotide comparisons of *C.bhatii* (GMBCC 1120, ex-type), *C.coffeae* (GMBCC 1130, ex-type), and *C.puerensis* (GMBCC 1113, ex-type) based on ITS, LSU, *tef*1-α, and *rpb*2; all of them were compared, excluding gaps.

Species	ITS (%)	LSU (%)	*tef*1-α (%)	*rpb*2 (%)
*C.puerensis* vs. *C.coffeae*	44/628(7.0%)	15/900(1.67%)	61/914 (6.65%)	118/1076 (10.97%)
*C.puerensis* vs. *C.bhatii*	42/484 (8.68%)	17/895 (1.9%)	70/924 (7.58%)	165/1076 (15.76%)
*C.coffeae* vs. *C.bhatii*	37/515 (7.18%)	12/901 (1.33%)	76/969 (7.84%)	172/1070 (16.07%)

#### ﻿ Camporesiomyces puerensis

Taxon classificationFungiTubeufialesTubeufiaceae

M.Y. Han &Tibpromma sp. nov.

CA44A220-C970-51C6-A7F7-91970FC49BE2

Index Fungorum: IF903590

Facesoffungi Number: FoF17677

Fig. 4

##### Etymology.

The name reflects the type location, “Pu’er” City, China.

##### Holotype.

GMB-W1121

##### Description.

***Saprobic*** on dead branches of *Coffeaarabica*. **Sexual morph**: Undetermined. **Asexual morph**: Hyphomycetous. *Colonies* on the natural substrate are superficial, solitary or clusters, hairy, yellow at apex velvety. *Mycelium* exposed on the surface of the substrate, with the exception of the roots. ***Conidiophores*** 52–176.5 × 2.8–5.6 μm (x̄ = 88.4 × 4 μm, *n* = 30), macronematous, mononematous, solitary or forming small clusters, smooth or occasionally verruculose, cylindrical, brown, flexuous, simple, unbranched, 3–13-septate, sometimes slightly constricted at the septa. ***Conidiogenous cells*** 6.8–26 × 2.3–4.3 μm (x̄ = 15 × 3.6 μm, *n* = 20), polyblastic, terminal, cylindrical to slightly tapering, denticulate, smooth, slightly curved, pale brown. ***Conidia*** 21.7–83 × 4–9.4 μm (x̄ = 43.3 × 6.3 μm, *n* = 30), solitary, acrogenous, cylindrical, or fusiform, sometimes slightly curved, 4–9-septate, subhyaline to yellow, hyaline at both rostrate ends.

##### Culture characteristics.

Conidia germinating on PDA within 24 h at 28 °C. Colony on PDA reaching 2 cm diam. after two weeks, circular or irregular, umbonate, with entire margin dark brown to black, reverse side displays predominantly black colonies with brown peripheral.

##### Material examined.

China • Yunnan Province, Pu’er City, Simao District (22°36'36"N, 101°0'14"E, 1189 m), on dead branches of *Coffeaarabica*, 8 August 2024, M.Y. Han & Tibpromma, (BG25 = GMB-W1121, holotype), ex-type GMBCC 1113, other living culture GMBCC 1114.

##### GenBank number.

GMBCC 1113 = ITS: PQ763356, LSU: PQ842541, *tef*1-α: PV388886, *rpb*2: PV388892 and GMBCC 1114 = ITS: PQ763357, LSU: PQ842542, *tef*1-α: PV388887*rpb*2: PV388893.

##### Notes.

In the concatenated phylogenetic analysis, our strain *Camporesiomycespuerensis* [GMBCC 1113 (ex-type), and GMBCC 1114] formed a sister branch with *C.coffeae* [GMBCC 1130 (ex-type), and GMBCC 1131] with 98% ML/1.00 PP bootstrap support (Fig. 1), both taxa formed an independent branch under *C.patagoniensis* (BBB MVB 573) with 70% ML/0.99 PP statistical support. Nucleotide comparisons between *C.puerensis* (GMBCC 1113) and *C.coffeae* (GMBCC 1130) are shown in Table 3. Morphologically, *C.patagoniensis* exhibits the sexual morph, so the morphological comparisons with our strain were unavailable. *Camporesiomycespuerensis* shares a similar conidial shape with *C.coffeae*, but it can be distinguished by its conidial pigmentation and dimensions. The conidia of *C.puerensis* are subhyaline to pale green, contrasting with the subhyaline to hyaline conidia of *C.coffeae* (Figs 3, 4). In addition, *C.puerensis* produces longer and broader conidia than *C.coffeae* (21.7–83 × 4–9.4 μm vs. 20–50 × 3.3–6.5 μm). *Camporesiomycespuerensis* also produces longer and slightly wider conidiophores than *C.coffeae* (52–176.5 × 2.8–5.6 μm vs. 43–97 × 2.8–4.5 μm) (Figs 3, 4). Hence, we describe *C.puerensis* as a new species based on both morphology and phylogeny.

## ﻿Discussion

In recent years, research on the diversity of microfungi on coffee plants has made significant progress, revealing their complexity and importance within the coffee ecosystem (dos Santos Gomes et al. 2024; Lu et al. 2025). These studies not only covered various ecological types, such as pathogens, endophytes, and saprophytes, but also emphasized the potential role of microfungi in coffee health and ecosystem function (Hyde et al. 2014; Bahram and Netherway 2022; Lu et al. 2022a; dos Santos Gomes et al. 2024). Pathogenic fungi are among the most widely studied microfungi in coffee ecosystems, while saprobic and endophytic fungi have received relatively less attention (Lu et al. 2022a; Poma-Angamarca et al. 2024; Lu et al. 2025). Saprophytic fungi play a crucial role in the coffee ecosystem functioning by decomposing organic matter and facilitating nutrient cycling (Dequn and Hyde 2001; Hyde et al. 2007). Some studies have suggested that the saprotrophic fungus *Phialomycesmacrosporus*, isolated from leaf litter, could potentially be used in the management of coffee halo blight in seedlings (Botrel et al. 2018). Therefore, studying the diversity of coffee saprophytic fungi is essential. By characterizing these fungi, we can identify key species that efficiently decompose organic matter, improve soil structure, suppress pathogens, and ultimately support more resilient and productive coffee ecosystems.

In this study, we introduce three new asexual species of *Camporesiomyces*, *viz.*, *C.bhatii*, *C.coffeae*, and *C.puerensis*, which were isolated from decaying coffee branches based on morphological and phylogenetic analyses. *Camporesiomyces* currently comprises three recognized species: *C.mali*, *C.patagonicus* (sexual morph), and *C.vaccinii* (asexual morph) (Carris 1989; Sanchez et al. 2012; Hyde et al. 2020). The three novel asexual species (*C.bhatii*, *C.coffeae*, *C.puerensis*) belonging to *Camporesiomyces* exhibit distinct morphological differences from the currently recognized taxa. Our three new *Camporesiomyces* species share similar morphological characteristics, including fusiform conidia and analogous conidiophores and conidiogenous cells, but differ primarily in their dimensional measurements. However, our three new species exhibit significant morphological differences from the asexual species *C.vaccinii. Camporesiomycesvaccinii* displays substantial morphological divergence, with spirally convolute conidia and conidiophores, characterized by multiple (1–3) percurrent proliferations (Carris 1989) (the detailed morphological distinctions are provided in Table 4). This discrepancy may be explained by geographical isolation (*C.vaccinii* occurring on *Vacciniumelliotii* stems in Georgia, USA) and host specificity (Carris 1989), suggesting environmental factors potentially drive morphological differentiation within the genus *Camporesiomyces*. The morphological similarity among our three species may, therefore, be attributed to their shared host and geographically proximate distribution.

**Table 4.** Morphological comparisons of asexual morphs in *Camporesiomyces*.

Species	Conidiophores	Conidiogenous cells	Conidia	Host/location	References
*C.bhatii* (GMB-W1176)	23–87.4 × 2.4–5.7 μm, brown, 2–8-septate, sometimes slightly constricted at the septa.	8.8–21.7 × 3–4.7 μm, cylindrical, slightly tapering, conspicuously denticulate on conidial secession, pale brown.	16–30 × 3.3–6.3 μm, obclavate or fusiform, 3–8-septate, subhyaline to pale brown.	Dead branches of *Coffealiberica*/China	This study
*C.coffeae* (GMB-W1181)	43–97 × 2.8–4.5 μm, dark brown, with longitudinal striations in the upper part, 2–9-septate, sometimes slightly constricted at the septa.	8–21 × 2.3–4.6 μm, cylindrical, with longitudinal striations, with several conspicuous denticles at apex, brown.	20–50 × 3.3–6.5 μm, guttules, cylindrical, fusiform, 3–7-septate, subhyaline or hyaline, obtuse or conical at both ends.	Dead branches of *Coffeaarabica*/China	This study
*C.puerensis* (GMB-W1121)	52–176.5 × 2.8–5.6 μm, brown, 3–13-septate, sometimes slightly constricted at the septa.	6.8–26 × 2.3–4.3 μm, cylindrical to slightly tapering, denticulate, smooth, slightly curved, pale brown.	21.7–83 × 4–9.4 μm, or fusiform, sometimes slightly curved, 4–9-septate, subhyaline to yellow, hyaline at both rostrate ends.	Dead branches of *Coffeaarabica*/China	This study
*C.vaccinii* (LMC 0043)	64–145 μm long, 4.2–5.0 μm wide at base, 2.5–3.3 μm wide at apex, simple or rarely branched, dark brown with paleapical cells, occasionally roughened, 4–10 septate, usually with l–2 percurrent proliferations.	1.3–2.7 × 1.0–1.3 μm, mono-or polyblastic, denticulate, denticles.	8.0–13.0 μm, conidial filament coiled l.5–l.75 times, 4–8-septate, rounded at apex and tapering to a truncate base, 2.0–4.0 μm, hyaline to pale brown, smooth-walled.	On stems of *Vacciniumelliotii*/America	Carris (1989)

We present descriptions, illustrations, and phylogenetic analysis results to validate and confirm the novelty of the three species: *C.bhatii*, *C.coffeae*, and *C.puerensis*. This is the first report of *Camporesiomyces* fungi associated with coffee, contributing three new asexual species to the genus *Camporesiomyces*. Our findings significantly enrich the fungal diversity of *Camporesiomyces* and hold substantial scientific value for enhancing our understanding of the fungal community inhabiting coffee hosts.

## Supplementary Material

XML Treatment for Camporesiomyces bhatii

XML Treatment for Camporesiomyces coffeae

XML Treatment for Camporesiomyces puerensis

## ﻿Additional information

### Author ORCIDs

Mei-Yan Han https://orcid.org/0009-0004-3705-5408

Samantha C. Karunarathna https://orcid.org/0000-0001-7080-0781

Li Lu https://orcid.org/0000-0003-0977-6414

De-Ge Zheng https://orcid.org/0009-0001-9199-6784

Nakarin Suwannarach https://orcid.org/0000-0002-2653-1913

Abdallah M. Elgorban https://orcid.org/0000-0003-3664-7853

Dong-Qin Dai https://orcid.org/0000-0001-8935-8807

Li-Juan Zhang https://orcid.org/0000-0002-3234-6757

Wan-Tong Zhao https://orcid.org/0009-0006-7298-0150

Ekachai Chukeatirote https://orcid.org/0000-0002-9968-5841

Saowaluck Tibpromma https://orcid.org/0000-0002-4706-6547

### Data availability

All of the data that support the findings of this study are available in the main text.
